# Diagnostic Performance of Microvascular Imaging for Detecting Histologically Confirmed Liver Fibrosis in Autoimmune Hepatitis: Comparison with Transient Elastography and Serum Biomarkers

**DOI:** 10.3390/diagnostics16132072

**Published:** 2026-07-02

**Authors:** Nazugum Ashimova, Aigul Raissova, Evgeniy Yenin, Rabiga Khozhamkul, Zhamilya Zholdybay, Maigul Shamshidinova, Takhmina Usenova, Andreas Teufel, Aigerim Mustapayeva, Alexander Nersesov

**Affiliations:** 1 Department of Gastroenterology, Asfendiyarov Kazakh National Medical University, Almaty 050012, Kazakhstan; nazugumashimova90@gmail.com (N.A.); maigul.1981@mail.ru (M.S.); paltakhunovat@gmail.com (T.U.); alexander.nersesov@gmail.com (A.N.); 2 Institute of Gastroenterology, Hepatology and Metabolism “Interna Clinic”, Almaty 050000, Kazakhstan; 3 National Scientific Center of Surgery Named After A. N. Syzganov, Almaty 050004, Kazakhstan; 4 Department of Health Policy and Public Health, Al-Farabi Kazakh National University, Almaty 050040, Kazakhstan; rabigaj@gmail.com; 5 Department of Internal Medicine A, University of Greifswald, 17489 Greifswald, Germany; andreas.teufel@med.uni-greifswald.de

**Keywords:** autoimmune hepatitis, microvascular imaging, transient elastography, liver fibrosis, APRI, FIB-4

## Abstract

**Background/Objectives**: Autoimmune hepatitis (AIH) is a chronic immune-mediated liver disease that may progress to cirrhosis and liver failure if not diagnosed early. Although liver biopsy remains the reference standard for fibrosis assessment, its invasive nature limits routine use. This study aimed to compare the diagnostic performance of ultrasound-based microvascular imaging (MVI), transient elastography (TE), and serum fibrosis indices (APRI and FIB-4) in patients with biopsy-confirmed AIH. **Methods:** Fifty-five patients with probable or definite AIH according to IAIHG criteria were included in the study. All patients underwent liver biopsy, and fibrosis stage was assessed using the METAVIR system. TE and MVI examinations were performed, and APRI and FIB-4 scores were calculated. Diagnostic performance was evaluated using AUROC, sensitivity, and specificity. Spearman correlation and logistic regression analyses were additionally performed. **Results**: The mean age of the patients was 49.2 years, and most patients were women. Cirrhosis was present in 58.2% of the cohort. TE demonstrated high diagnostic accuracy, whereas FIB-4 showed moderate performance and APRI demonstrated limited utility. MVI achieved the highest diagnostic performance, with AUROC values of 0.99 for significant fibrosis and 0.97 for cirrhosis. MVI showed the strongest correlation with histological fibrosis stage (r = 0.916, *p* < 0.001), followed by TE (r = 0.907, *p* < 0.001). MVI was strongly associated with histologically confirmed cirrhosis (OR 16.7, 95% CI 2.36–118.2, *p* = 0.004). **Conclusions**: MVI demonstrates diagnostic performance comparable to TE and may represent a promising adjunctive non-invasive imaging biomarker for fibrosis assessment in AIH. Larger multicenter studies are required for external validation before routine clinical implementation.

## 1. Introduction

Autoimmune hepatitis (AIH) is an uncommon, immune-mediated inflammatory liver disease characterized by circulating autoantibodies, elevated immunoglobulin G (IgG) levels, and distinct histological features [1]. Globally, the annual incidence and prevalence are estimated at 1.37 and 17.44 cases per 100,000 population, respectively, with prevalence rates of 12.99 in Asians, 19.44 in Europeans, and 22.80 in Americans per 100,000 people [2]. Improved diagnostic methods and greater clinical awareness have likely increased the detection of AIH, reducing the number of missed cases, including in adult men [3].

According to the Clinical Protocol of the Ministry of Health of the Republic of Kazakhstan, the incidence of AIH is approximately 1.9 cases per 100,000 population per year [4]. However, this figure may underestimate the true prevalence in the region, warranting further epidemiological investigation.

The diagnosis of AIH is challenging due to a lack of pathognomonic criteria and a wide spectrum of clinical, serological, and morphological presentations. This heterogeneity often delays the initiation of immunosuppressive therapy, which can adversely affect patient prognosis [5]. Liver biopsy is the primary method for confirming the diagnosis and is recommended to guide treatment decisions. However, as an invasive procedure, it carries a risk of complications [6], limiting its utility as a routine method for monitoring fibrosis and cirrhosis [7].

In clinical practice, several non-invasive markers are used to assess liver fibrosis. Inexpensive serum-based tests include the AST-to-platelet ratio index (APRI) and the Fibrosis-4 (FIB-4) index. While these markers can be used to detect cirrhosis in the context of chronic viral hepatitis and metabolic dysfunction-associated steatotic liver disease (MASLD) [8], their ability to identify early-stage fibrosis in patients with AIH remains uncertain [9].

Transient elastography (TE, FibroScan) has gained widespread acceptance as a validated non-invasive method for staging liver fibrosis [10]. Since fibrosis progression is a key determinant of prognosis in AIH, it should be assessed morphologically. However, repeat biopsies are often avoided, and clinicians frequently rely on systematic liver stiffness measurement (LSM) via TE instead [11]. TE also analyzes a much larger portion of the liver parenchyma, exceeding the volume of a typical biopsy sample by a factor of 100 [12]. Nevertheless, the accuracy of TE in AIH remains debated, as LSM values can be falsely elevated by the moderate-to-severe necroinflammatory activity characteristic of the disease [13].

Persistent liver inflammation in AIH can activate stellate cells and promote the accumulation of extracellular matrix components, which contribute to the progression of fibrosis and cirrhosis. In addition to structural changes in the liver parenchyma, fibrosis can lead to microvascular remodeling, including capillarization of vessels, deformation and loss of vascular branches, and disruption of intrahepatic blood flow [14]. Since these changes affect the liver’s microvascular network, they can potentially be detected using modern ultrasound technologies such as MVI.

Ultrasound methods based on the Doppler effect, predominantly known as microvascular imaging (MVI), enable visualization of low-velocity microvascular blood flow. By preserving signals from slow-flow vessels, MVI reduces image artifacts and provides detailed visualization of small intrahepatic vascular structures. MVI detects vascular and perfusion abnormalities that may not be fully captured by measuring liver stiffness or serum-based fibrosis indices. Therefore, MVI may provide complementary information on fibrosis-related vascular remodeling and may serve as a potential imaging biomarker for fibrosis staging in AIH. Despite growing interest in MVI in chronic liver diseases, data regarding its application for fibrosis staging in AIH remain scarce.

Although no previous studies have directly evaluated MVI for fibrosis staging in AIH, research in other chronic liver diseases suggests that microvascular and perfusion parameters change during fibrosis progression.

To our knowledge, this is the first biopsy-validated study evaluating MVI for fibrosis staging specifically in autoimmune hepatitis and the first report from Central Asia.

This study aimed to evaluate and compare the diagnostic accuracy of MVI, TE, and the serum indices APRI and FIB-4 for staging liver fibrosis in a cohort of patients with morphologically confirmed AIH in Kazakhstan.

## 2. Materials and Methods

### 2.1. Patient Population

This prospective single-center study was conducted at the Institute of Gastroenterology, Hepatology, and Metabolism of the Clinical Center of KazNMU named after S.D. Asfendiyarov, Almaty, Kazakhstan. Adult patients with a probable or confirmed diagnosis of AIH according to the simplified criteria of IAIHG [15] were consecutively enrolled from May 2025 to March 2026 and underwent liver biopsy. The inclusion criteria were age ≥18 years and a confirmed diagnosis of AIH based on serological and histological data. Only patients with complete clinical, laboratory, and histological data, as well as transient elastography and microvascular visualization results, were included in the final analysis.

The exclusion criteria included the presence of viral hepatitis, significant alcohol consumption (>30 g/day for men and >20 g/day for women), MASLD, primary biliary cholangitis, primary sclerosing cholangitis, drug-induced liver injury, Wilson’s disease, hemochromatosis, or hepatocellular carcinoma, and incomplete clinical and laboratory data.

At the time of biopsy, the treatment status of the patients in this cohort was heterogeneous. Three patients (5.5%) were prescribed prednisolone (for less than one month) based on clinical manifestations. The remaining 52 patients (94.5%) were taking ursodeoxycholic acid before diagnosis.

The use of UDCA before diagnosis verification reflected empirical therapy at the diagnostic search stage in patients with unexplained cytolysis and was not associated with the presence of overlapping syndromes (AIG-PBCH; AIG-PSH). The diagnosis of AIG was confirmed by clinical, immunological, and histological criteria, allowing for the formation of a diagnostically homogeneous study cohort.

**Figure 1:**
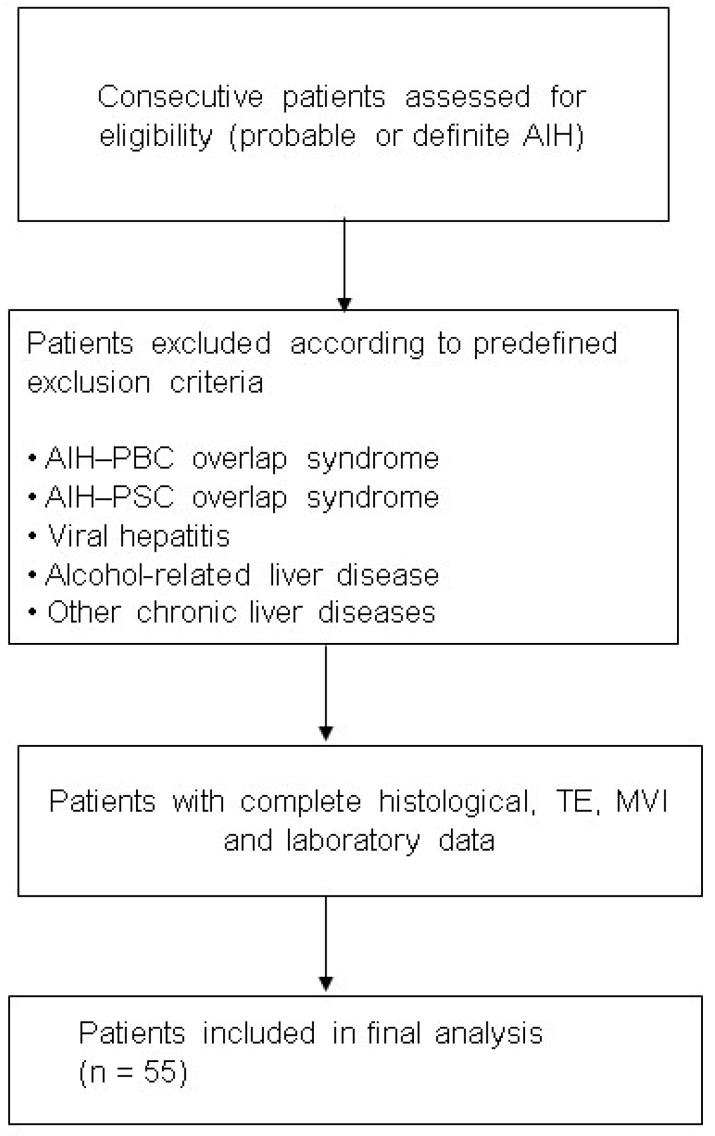
Flow diagram of patient selection and inclusion in the final analysis.

The patient selection process, eligibility assessment, and inclusion in the final analysis are summarized in Figure 1.

### 2.2. Liver Biopsy

Representative liver tissue samples were obtained using an 18-gauge biopsy needle. The biopsy specimens were fixed in 10% neutral buffered formalin for standard histological processing. Adequate samples were defined as having a length of at least 1.5 cm and containing at least 8–10 portal tracts, in line with international recommendations [16]. Fibrosis stage and histological activity were assessed using the METAVIR scoring system (F0: no fibrosis to F4: cirrhosis) [17]. The modified Ishak score was also used [18]. Key morphological criteria for AIH included lymphoplasmacytic infiltration, interface hepatitis, and emperipolesis.

### 2.3. Ultrasound with Microvascular Imaging (MVI)

B-mode ultrasound was performed using a General Electric HealthCare Systems (Milwaukee, WI, USA) with a 3.5 MHz convex transducer. The examination was conducted by a single operator with 19 years of experience. The liver structure was assessed 2 cm from the capsule in the anterior segments. MVI was used to evaluate vascular morphology and identify signs of fibrosis, which were graded from 0 to IV based on a previously established classification of vascular alterations (Figure 1):

- Grade I: Normal or minimal loss of tapering and branching of the vascular tree.
- Grade II: Tortuosity of the main branches and pronounced tortuosity of distal branches.
- Grade III: Reduced number of small distal branches (“pruned tree” appearance).
- Grade IV: Reduced number and visualization of larger branches [19].

Blinding. To minimize observer bias, the operator performing MVI examinations was blinded to histopathological fibrosis staging, transient elastography measurements, laboratory results, and clinical data during image acquisition and interpretation.

### 2.4. Transient Elastography and Serum Biomarkers

TE was performed before biopsy using a FibroScan Expert 630 (FibroScan^®^, Echosens, Paris, France). LSM was measured in the right lobe of the liver, with at least 10 valid measurements, a success rate ≥ 60%, and an interquartile range (IQR)/median ratio ≤ 30% considered as providing a reliable result. The APRI and FIB-4 indices were calculated using standard formulas [20,21], and individual continuous kPa measurements were recorded.

### 2.5. Statistical Analysis

Statistical analysis was performed using IBM SPSS Statistics version 29.0 (IBM Corp., Armonk, NY, USA) and R sorftware (version 4.3.2; R Foundation for Statistical Computing, Vienna, Austria). The diagnostic performance of MVI, TE, APRI, and FIB-4 was evaluated by calculating the AUROC. Optimal cut-off values for detecting fibrosis stages F ≥ 2, F ≥ 3, and F4 were determined using the Youden index. Sensitivity, specificity, positive predictive value (PPV), negative predictive value (NPV), and likelihood ratios (LR+ and LR−) were calculated for each test. Spearman correlation analysis and univariate logistic regression analysis were additionally performed to evaluate associations between non-invasive fibrosis assessment methods and histologically confirmed cirrhosis. To address potential instability in diagnostic accuracy estimates arising from the small sample size in certain fibrosis categories (e.g., F3), we performed bootstrapping with 2000 resamples to calculate robust 95% confidence intervals (CIs) for the AUROC values. This resampling technique provides a more robust estimation of diagnostic performance by mitigating the impact of sample size limitations.

Generative artificial intelligence (GenAI) was not used in this study for writing the manuscript, data analysis, interpretation of results, or creation of graphical materials.

## 3. Results

This study included 55 patients with a mean age of 49.2 ± 14.5 years; 47 (85.5%) were women and 8 (14.5%) were men. According to the simplified IAIHG criteria, 11 (20%) patients were diagnosed with probable AIH and 44 (80%) with definite AIH. The cohort was predominantly Asian (83.6%), with Caucasians accounting for nine patients (16.4%) (Table 1).

The immunological profile of 44 (80%) patients was characterized by dominant ANA positivity, while ASMA antibodies were not detected in the study cohort. Elevated IgG was documented in 35 (63.6%) patients, whereas elevated gamma globulins were found in fewer than half of the patients, specifically in 24 patients (43.6%).

Morphological analysis revealed a marked shift toward advanced fibrosis stages. Cirrhosis (F4) according to the METAVIR scale was identified in 32 (58.2%) patients. Early-stage fibrosis (F0–F1) was relatively uncommon, present in 14 (25.5%) patients, while significant fibrosis (F2–F3) was determined in 9 (16.4%) patients. The histological activity index demonstrated moderate activity (A2) in 27 (49.1%) patients and minimal activity (A1) in 28 (50.9%) patients. No cases of absent activity (A0) or severe activity (A3) were observed in the study group. Lymphoplasmacytic infiltration was present in 100% of patients, interface hepatitis in 52 (94.5%), and emperipolesis in 49 (89.1%) of cases.

Table 2 presents the results of the comparison between non-invasive fibrosis markers and fibrosis stages according to the METAVIR scale. The analysis revealed significant differences in the diagnostic sensitivity of the various non-invasive methods.

The APRI index showed poor concordance with histological staging. In 22 (40%) patients, no signs of fibrosis (F0) were detected, while advanced fibrosis (F4) was diagnosed in only 8 (14.5%) patients. The FIB-4 index demonstrated a more balanced distribution of fibrosis stages, with the F0 and F4 stages detected in similar frequency in 15 patients (27.3%).

FibroScan showed improved performance, detecting advanced fibrosis (F4) in 22 (40%) patients and intermediate stages (F2–F3) in 19 (34.5%) patients, which more closely approximated the histological findings. MVI showed close agreement with histological findings for cirrhosis (F4), identifying this stage in 30 (54.5%) patients, which was substantially closer to the morphological data.

Histological examination, the gold standard for fibrosis staging, revealed that the majority of patients, specifically 32 (58.2%), had cirrhosis (F4). Therefore, both APRI and FIB-4 substantially underestimated the prevalence of advanced fibrosis, whereas transient elastography and MVI demonstrated the greatest concordance with biopsy findings.

Table 3 presents the distribution of non-invasive fibrosis assessment methods across each fibrosis stage according to the METAVIR scale. Using the Kruskal–Wallis test, we identified significant differences across fibrosis stages when comparing APRI values (*p* < 0.001), FIB-4 values (*p* < 0.001), and liver stiffness measurement (LSM) values (*p* < 0.001).

The non-invasive fibrosis markers APRI, FIB-4, and LSM demonstrated significantly higher values in patients with advanced fibrosis (METAVIR F4) compared with earlier fibrosis stages (METAVIR F0–F3). No statistically significant differences were observed with respect to patient age.

**Figure 2:**
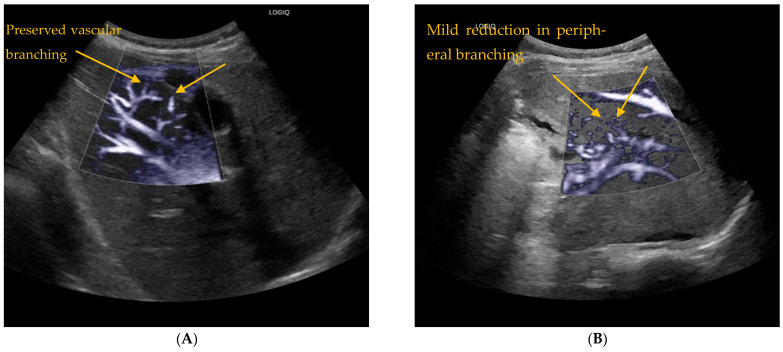

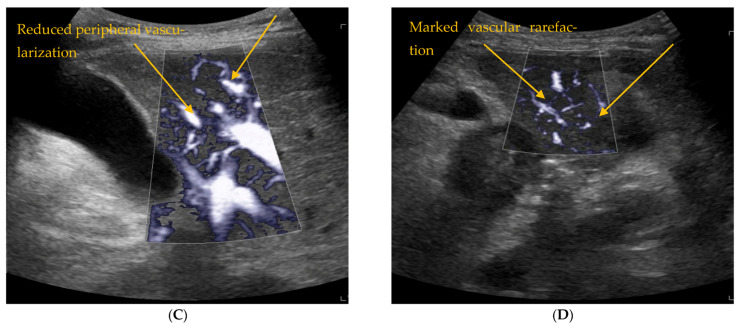
Representative MVI images corresponding to METAVIR fibrosis stages F1–F4 in patients with autoimmune hepatitis. Progressive reduction in peripheral vascular branching and increasing vascular architectural distortion can be observed with advancing fibrosis stage. (**A**) F1; (**B**) F2; (**C**) F3; (**D**) F4. Yellow arrows indicate areas of vascular branching.

Figure 2 illustrates the distribution of APRI (*p* = 0.015), FIB-4 (*p* < 0.001), and LSM (*p* < 0.001) values according to METAVIR fibrosis stage. All markers demonstrated progressive increases in values with advancing fibrosis stages. MVI demonstrated the strongest correlation with histological fibrosis stage, closely followed by liver stiffness measurement (LSM).

Table 4 presents the diagnostic performance characteristics of non-invasive methods for assessing fibrosis, including area under the receiver operating characteristic curve (AUROC), sensitivity, and specificity, with threshold values for F2, F3, and F4 according to the METAVIR scale.

The FIB-4 index demonstrated moderate diagnostic accuracy in early fibrosis stages (AUROC 0.68–0.75), with high specificity for F4 (96%) but low sensitivity, indicating limited utility for detecting cirrhosis.

The APRI index demonstrated the lowest diagnostic performance among the evaluated methods, with AUROC values of 0.64 for significant fibrosis and 0.59 for advanced fibrosis.

FibroScan demonstrated high diagnostic efficacy for detecting significant and advanced fibrosis, with AUROC values of 0.98 for F ≥ 2 and 0.95 for F ≥ 3. For cirrhosis (F ≥ 4), the AUROC was 0.84, indicating moderate accuracy. The sensitivity of TE was 98% for F ≥ 2, 94% for F ≥ 3, and 100% for F ≥ 4, while the specificity for these respective stages was 94%, 96%, and 100%.

MVI demonstrated the highest diagnostic performance, with AUROC values of 0.99 for both F ≥ 2 and F ≥ 3 and 0.97 for F ≥ 4, sensitivity of 94–98%, and specificity of 100%. These findings suggest that MVI may be useful for non-invasive fibrosis assessment in this cohort.

**Figure 3:**
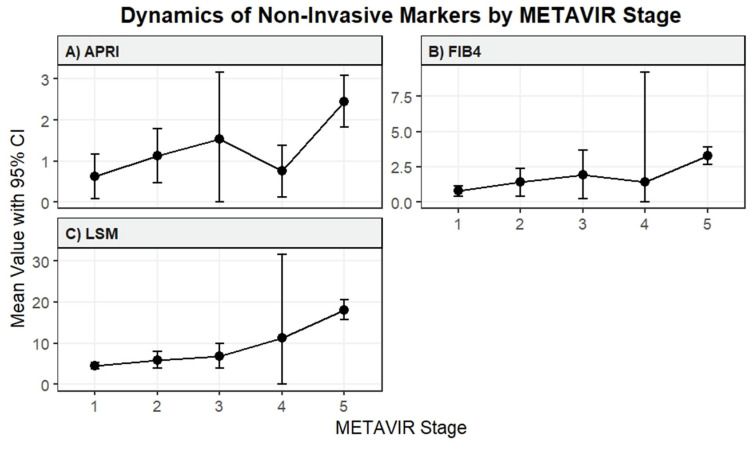
Correlation between non-invasive fibrosis assessment methods and METAVIR fibrosis stages. Distribution of non-invasive fibrosis assessment parameters according to METAVIR fibrosis stage. The box plots demonstrate progressive increases in APRI (**A**), FIB-4 (**B**), and liver stiffness measurement (LSM) values (**C**) with advancing fibrosis stage, with MVI showing the strongest overall correlation with histological fibrosis severity. Horizontal lines within the boxes represent median values, and box boundaries indicate the interquartile range.

Figure 3 presents the ROC analysis comparing the diagnostic performance of all non-invasive methods. The analysis demonstrated that MVI and TE exhibited the best diagnostic performance for detecting all fibrosis stages, with AUROC values exceeding 0.94, coupled with high sensitivity and specificity values.

**Figure 4:**
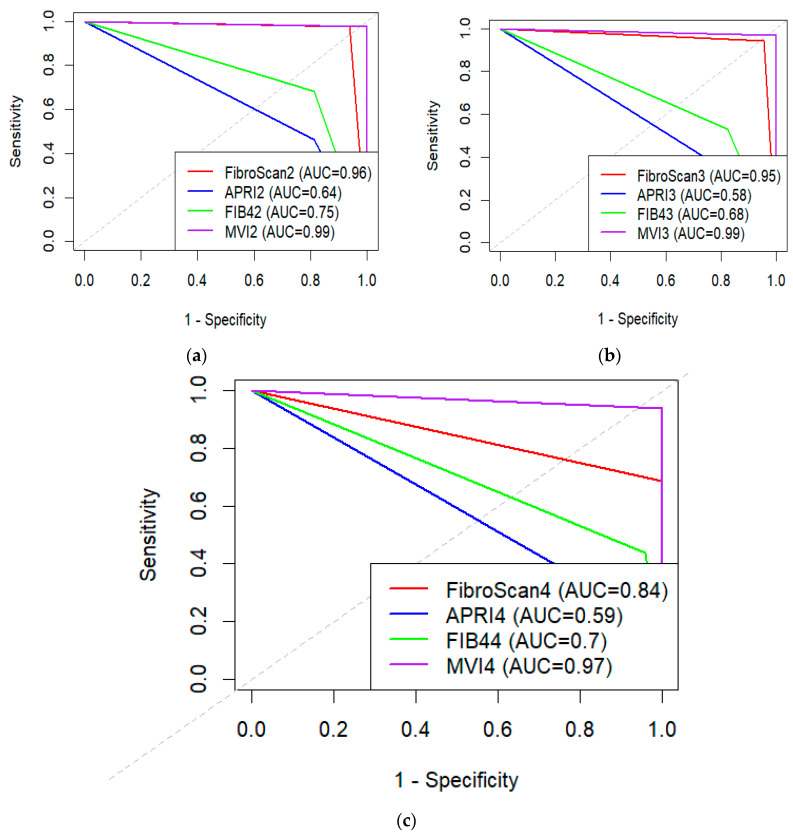
Receiver operating characteristic (ROC) curves comparing the diagnostic performance of non-invasive fibrosis assessment methods for detecting significant fibrosis (F ≥ 2), advanced fibrosis (F ≥ 3), and cirrhosis (F4) according to METAVIR staging. MVI and TE show higher diagnostic accuracy than serum biomarkers across all fibrosis stages, whereas APRI and FIB-4 show substantially lower performance, particularly for advanced fibrosis and cirrhosis. (**a**) Significant fibrosis (F ≥ 2); (**b**) Advanced fibrosis (F ≥ 3); (**c**) Cirrhosis (F4).

The FIB-4 index demonstrated a more balanced distribution across fibrosis stages; however, its diagnostic efficacy (AUROC 0.75) was inferior to both MVI and TE, particularly for advanced fibrosis stages (Figure 4).

The APRI index showed the lowest diagnostic performance among all methods evaluated, as confirmed by the low AUROC (0.64), sensitivity, and specificity (Figure 4).

Spearman correlation analysis demonstrated that MVI had the strongest correlation with histological fibrosis stage (r = 0.916, *p* < 0.001), followed closely by TE (r = 0.907, *p* < 0.001). Serum biomarkers, specifically FIB-4 (r = 0.627, *p* < 0.001) and APRI (r = 0.544, *p* < 0.001), demonstrated substantially weaker correlations.

The results of the correlation and logistic regression analyses are presented in Table 5. In univariate logistic regression analysis, MVI demonstrated a strong association with histologically confirmed cirrhosis (OR: 16.7, 95% CI: 2.36–118.2, *p* = 0.004). TE, APRI, and FIB-4 also showed significant association with cirrhosis, whereas age and IgG levels were not significant predictors.

**Table 5:** Correlation and logistic regression analyses of non-invasive fibrosis assessment methods.

Variable	Spearman r	*p*-Value	OR	95% CI	*p*-Value
MVI	0.916	<0.001	16.7	2.36–118.2	0.004
FibroScan	0.907	<0.001	3.45	1.48–8.05	0.004
FIB-4	0.627	<0.001	1.97	1.33–2.91	<0.001
APRI	0.544	<0.001	2.38	1.41–4.02	0.001

Multivariable modeling demonstrated substantial collinearity between MVI and TE, suggesting overlapping diagnostic information between these modalities.

## 4. Discussion

The present study suggests that MVI may provide high diagnostic performance for staging liver fibr osis in AIH and shows diagnostic characteristics comparable to TE when validated against histological assessment. Among all evaluated non-invasive methods, MVI demonstrated the strongest correlation with fibrosis stage and the highest diagnostic accuracy for advanced fibrosis and cirrhosis.

The strong correlation between MVI and histological fibrosis stage suggests that fibrosis-related vascular remodeling may represent a potential imaging biomarker of fibrosis progression in AIH. Unlike TE, which primarily reflects tissue stiffness, MVI may additionally capture microvascular architectural distortion associated with progressive fibrosis.

This study is the first to describe the clinical and immunological characteristics of AIH and to compare non-invasive fibrosis assessment methods (MVI, TE, APRI, and FIB-4) with histological findings in a Kazakhstan population.

The predominance of middle-aged women, high frequency of antinuclear antibodies, variability of the serological profile, and levels of IgG and gamma globulins are consistent with data from international cohort studies and clinical guidelines [12,22,23,24].

Although anti-ASMA antibodies were not detected in our cohort, their detection was performed using indirect immunofluorescence, which is the recommended screening method for autoimmune liver diseases. Therefore, the absence of ASMA positivity is unlikely to be related to laboratory testing characteristics. This finding may reflect serological variability of AIH, features of the studied cohort, or a relatively small sample size and requires further investigation in larger patient populations.

Regarding the morphological characteristics of AIH, all patients demonstrated minimal to moderate inflammatory activity (A1–A2), with no cases showing absent activity (A0) or severe activity (A3). These histological findings are characteristic of chronic AIH progression, as documented in previous studies emphasizing that the degree of inflammatory activity does not always correlate with fibrosis stage, and severe histological activity (A3) is more commonly observed in acute or fulminant AIH presentations. Despite the variability of serological markers, the presence of characteristic histological features—including plasmocytic infiltration (100%), interface hepatitis (94.5%), and emperipolesis (89.1%)—confirms morphologically definite AIH and underscores the indispensable role of liver biopsy in AIH diagnosis. In our cohort, IST was initiated before biopsy in three patients. Due to the extremely small number of patients receiving IST, analysis by treatment response was not possible. However, the negative correlation between aminotransferase, IgG levels, and disease duration in patients without cirrhosis requires further analysis.

The high prevalence of cirrhosis (F4), observed in 58.1% of the patients, likely reflects delayed referral to specialized care and the frequently insidious course of AIH during its early stages. Limited awareness of AIH among primary care physicians may contribute to delayed immunological and histological evaluation in patients presenting with unexplained cytolytic syndrome. In addition, the absence of a unified national AIH registry in Kazakhstan may hinder timely referral and systematic disease monitoring. Collectively, these factors highlight the clinical relevance of the present study and underscore the need for earlier diagnosis of AIH.

When interpreting the diagnostic effectiveness of MVI and TE, it is important to consider the re-encoding of patients with liver cirrhosis. This re-encoding could have contributed to an overestimation of diagnostic accuracy compared with a broader population of patients with AIH characterized by a more even distribution of early, intermediate, and advanced stages of fibrosis.

Our study demonstrated that FibroScan has high diagnostic accuracy for assessing fibrosis stages in patients with AIH, as confirmed by AUROC values exceeding 0.94 for F ≥ 2 and F ≥ 3. The reliability of TE confirms the clinical significance of this method for identifying advanced fibrosis stages, with performance comparable to liver biopsy findings [13].

Discrepancies in fibrosis staging between biopsy and TE may be attributed to the limited volume of biopsy material used in TE, which may not reflect the true extent of diffuse liver injury, whereas FibroScan evaluates a substantially larger portion of liver tissue [25]. Therefore, the discordance between FibroScan and biopsy may reflect the limited representativeness of biopsy; however, this does not diminish the clinical value of TE for detecting severe fibrosis and cirrhosis.

The serum indices APRI and FIB-4 demonstrated low sensitivity and specificity, which could be explained by their original validation in viral hepatitis and reduced applicability in autoimmune liver diseases. The AUROC values for APRI in diagnosing various fibrosis stages were consistently below 0.7, indicating low diagnostic significance. Similar results have been demonstrated in other studies, emphasizing the limited utility of these markers in AIH diagnosis [12,26]. In our study, APRI showed lower efficacy compared with other methods; this is corroborated by data from a meta-analysis [26] and systematic review [8]. Both APRI and FIB-4 demonstrated suboptimal diagnostic efficacy for detecting significant and advanced fibrosis in patients with autoimmune hepatitis [9].

Ultrasound findings using the MVI technique for hepatic fibrosis have been studied by Ayşe Özlem Balık et al., who demonstrated that MVI may serve as an alternative to liver biopsy in severe hepatic fibrosis [27]. MVI has proven effective in detecting early-stage fibrosis in chronic liver diseases, particularly in hepatitis C, metabolic dysfunction-associated fatty liver disease (MASLD), and alcoholic liver disease [28].

Although these findings were obtained in patients with chronic liver diseases other than AIH, they support the concept that fibrosis-associated microvascular alterations can be detected by MVI, which may have diagnostic value across different etiologies of liver fibrosis.

Recent research findings confirm the diagnostic importance of non-invasive methods for assessing fibrosis and monitoring the course of chronic liver diseases. In a recent prospective study of patients with alcohol-related cirrhosis, fecal microbiota transplantation was associated with a statistically significant reduction in liver stiffness according to TE, as well as a decrease in systemic inflammation and a reduction in the severity of hepatic encephalopathy. Liver parameters demonstrated higher sensitivity to disease dynamics over a short observation period compared to APRI and FIB-4 [29]. Despite the different nature of the disease, these data further confirm the clinical significance of transient elastography as a non-invasive method for assessing fibrosis. Our results suggest that MVI can be used as an additional imaging method alongside TE for evaluating fibrosis in patients with AIH.

In our study, MVI demonstrated diagnostic performance comparable to TE while directly reflecting fibrosis-related microvascular remodeling in AIH. The strong correlation between MVI and histological fibrosis stage suggests that vascular architectural distortion may represent an important imaging biomarker of fibrosis progression. These findings support the potential role of MVI as a complementary non-invasive method for fibrosis assessment in AIH. Nevertheless, multicenter studies with external validation are required before routine clinical implementation. The stronger correlation between MVI features and histological fibrosis stage may be linked to the capability of MVI to represent microvascular architectural changes connected with hepatic fibrogenesis, including reduction in peripheral vascular branching and sinusoidal remodeling, which are characteristic findings of progressive liver fibrosis [30].

While TE (FibroScan) assesses the mechanical stiffness of the liver, MVI reflects vascular changes associated with fibrosis. These two methods may be considered complementary rather than competitive. Our findings suggest the diagnostic value of MVI and TE, with AUROC values reaching 0.99 and 0.98 for significant fibrosis, respectively. However, these high AUROC values must be interpreted cautiously within the context of the study’s cohort composition. Specifically, 58.2% of our patients presented with METAVIR F4 fibrosis (cirrhosis). This high prevalence of advanced disease introduces a potential spectrum bias, where diagnostic tests often perform better in cohorts with a higher proportion of severe cases compared to cohorts with a more balanced distribution across all disease stages. Consequently, the reported AUROC values may partly reflect the predominance of advanced fibrosis rather than equal discrimination across all early and intermediate fibrosis stages. Furthermore, the limited number of patients in certain intermediate fibrosis categories, particularly the F3 stage (*n* = 2, 3.6%), reduces the statistical stability of our diagnostic estimates for advanced fibrosis (≥F3). To mitigate this limitation and support the robustness of our findings, we incorporated a bootstrapping approach (2000 resamples) to calculate the 95% confidence intervals for the AUROC values. While bootstrapping confirms the high diagnostic utility of MVI and TE within this cohort, the small number of patients in intermediate stages remains a limitation. Future studies with larger, more evenly distributed cohorts are necessary to validate the performance of MVI across the full spectrum of fibrosis stages without the influence of spectrum bias.

The potential added value of combining TE and MVI should be evaluated in future prospective studies.

Liver stiffness measurements may depend not only on the level of fibrosis but also on the degree of non-inflammatory activity. Hartl and colleagues [31] demonstrated that inflammation activity significantly affects liver stiffness, which can lead to an overestimation of the fibrosis stage. In our cohort, most patients exhibited mild to moderate inflammatory activity (A1–A2), which could have reduced but not completely eliminated the potential influence of inflammation on the results of non-invasive fibrosis assessment. The impact of inflammatory activity on MVI results remains unclear and requires further study.

This study has several limitations. First, the relatively small sample size (*n* = 55) might have limited statistical power and could lead to overestimation of diagnostic accuracy. The exceptionally high AUROC values observed in this study should therefore be interpreted cautiously given the relatively small cohort size and predominance of advanced fibrosis stages. Second, the study cohort demonstrated an unequal distribution of fibrosis stages with a predominance of patients with cirrhosis, which may have contributed to spectrum bias and overestimation of diagnostic performance. Third, all MVI examinations were performed by a single experienced operator, limiting assessment of interobserver reproducibility. We acknowledge that intraobserver and interobserver variability analyses were not performed. As MVI assessment relies on visual interpretation of vascular morphology, the method remains operator-dependent. Future multicenter studies should evaluate the reproducibility and generalizability of the proposed grading system. Fourth, this was a single-center study, which limits the generalizability of the findings to other patient populations.

Finally, antibodies to soluble liver antigen (anti-SLA) were not routinely measured in our study due to limited availability in clinical practice in Kazakhstan. Given their potential association with disease severity, including anti-SLA in future studies may provide additional prognostic value.

## 5. Conclusions

TE is a reliable non-invasive method for staging liver fibrosis in patients with AIH, demonstrating superiority over serum biomarkers such as APRI and FIB-4. In this study, MVI was found to demonstrate diagnostic performance comparable to TE and may represent a promising adjunctive non-invasive imaging biomarker for fibrosis assessment in AIH. However, external validation in larger multicenter cohorts is required before routine clinical implementation.

## Figures and Tables

**Table 1:** Characteristics of AIH patients included in the study.

Patient Characteristics	Value
Total, *n*	55
Female, *n* (%)	47 (85.5)
Male, *n* (%)	8 (14.5)
Mean age in years, M ± SD	49.20 ± 14.47
Ethnicity	
Asians	46 (83.6)
Caucasians	9 (16.4)
ANA, *n* (%)	44 (80)
IgG > 1.1 ULT, *n* (%)	35 (63.6)
Gamma globulin, *n* (%)	24 (43.6)
Hepatic inflammatory activity, *n* (%)	
A0	0
A1	28 (50.9)
A2	27 (49.1)
A3	0
Fibrosis stage, *n* (%)	
F0	6 (10.9)
F1	8 (14.6)
F2	7 (12.7)
F3	2 (3.6)
F4	32 (58.2)
Morphological characteristics, *n* (%)	
Lymphocytic/plasma cell infiltration	55 (100)
Interface hepatitis	52 (94.5)
Emperipolesis	49 (89.1)

**Table 2:** Distribution of fibrosis stages according to histology, MVI, FibroScan, APRI, and FIB-4.

Fibrosis Stage	Histology (*n*, %)	MVI (*n*, %)	FibroScan (*n*, %)	FIB-4 (*n*, %)	APRI (*n*, %)
F0	6 (10.91)	5(9.1)	7 (12.73)	15 (27.27)	24 (43.64)
F1	8 (14.54)	9 (16.36)	7 (12.73)	10 (18.18)	10 (18.18)
F2	7 (12.73)	7 (12.73)	8 (14.54)	9 (16.36)	9 (16.36)
F3	2 (3.64)	4 (7.27)	11 (20.0)	6 (10.91)	4 (7.27)
F4	32 (58.18)	30 (54.54)	22 (40.0)	15 (27.28)	8 (14.55)
Total	55 (100)	55 (100)	55 (100)	55 (100)	55 (100)

Note: Histology based on METAVIR staging was used as the reference standard. Overall comparison of fibrosis stage distributions across methods was significant (Kruskal–Wallis test, *p* < 0.001).

**Table 3:** Comparison of non-invasive fibrosis markers between non-cirrhotic (F0–F3) and cirrhotic (F4) patients.

Parameter	Non-Cirrhotic Patients (F0–F3, *n* = 23)	Cirrhotic Patients (F4, *n* = 32)	*p*-Value
Age	45 (38–56)	57 (42–65)	0.191
APRI	0.7 (0.5–1.6)	2.75 (1.75–4.5)	<0.01
FIB-4	1.24 (0.8–2.03)	4.01 (2.17–5.17)	<0.001
FibroScan	7.9 (5.7–11.7)	20.05(17.7–24.5)	<0.001

**Table 4:** Diagnostic performance of MVI, FibroScan, APRI, and FIB-4 for detecting significant fibrosis (F ≥ 2), advanced fibrosis (F ≥ 3), and cirrhosis (F4).

Fibrosis Stage	Method	AUROC (95% CI)	Sensitivity (%)	Specificity (%)	Cut-Off Value	PPV (%)	NPV (%)
F ≥ 2	MVI	0.99 (0.96–1.00)	97.6	100.0	N/A	97.6	87.5
	FibroScan	0.95 (0.87–1.00)	97.6	92.9	7.1	97.6	92.9
	FIB-4	0.77 (0.65–0.88)	68.3	85.7	1.4	96.7	20.0
	APRI	0.66 (0.54–0.77)	46.3	85.7	1.75	97.7	18.6
F ≥ 3	MVI	0.99 (0.96–1.00)	97.1	100.0	N/A	100	95.7
	FibroScan	0.95 (0.88–1.00)	94.1	95.2	8.5	97.0	90.9
	FIB-4	0.69 (0.58–0.80)	52.9	85.7	1.58	95.2	38.2
	APRI	0.60 (0.50–0.70)	29.4	90.5	0.9	96.2	33.0
F4 (Cirrhosis)	MVI	0.97 (0.92–1.00)	93.8	100	N/A	96.8	92.3
	FibroScan	0.84 (0.77–0.92)	68.8	100	9.5	95.7	70.6
	FIB-4	0.70 (0.60–0.80)	43.8	95.7	1.58	93.3	50.0
	APRI	0.59 (0.50–0.67)	21.9	95.7	0.9	87.5	42.6

Note: Cut-off values for microvascular imaging (MVI) are denoted as ‘N/A’ because MVI was evaluated using an ordinal scale (Grades 0–IV) based on morphological vascular alterations, rather than as a continuous numeric variable. Diagnostic thresholds for MVI rely on grade progression rather than a specific numerical cut-off. Cut-off values for transient elastography (FibroScan) in kilopascals (kPa) were determined using the Youden index as follows: 7.1 kPa for detecting significant fibrosis (F ≥ 2), 8.5 kPa for detecting advanced fibrosis (F ≥ 3), and 9.5 kPa for detecting cirrhosis (F4). Bootstrap analysis with 2000 resamples was performed to calculate robust 95% confidence intervals for all AUROC values. AUROC, area under the receiver operating characteristic curve; PPV, positive predictive value; NPV, negative predictive value; CI, confidence interval; N/A, not applicable.

## Data Availability

The data presented in this study are available from the corresponding author upon reasonable request. Because this study involves clinical data from human participants, the datasets are not publicly available due to ethical and privacy restrictions. Access to de-identified data may be provided to qualified researchers, subject to Institutional Review Board approval and applicable data-sharing regulations.

## Abbreviations

The following abbreviations are used in this manuscript:

AIH	Autoimmune hepatitis
ALP	Alkaline phosphatase
ALT	Alanine aminotransferase
ANA	Antinuclear antibodies
Anti-HCV	Antibodies against hepatitis C virus
APRI	AST-to-platelet ratio index
AST	Aspartate aminotransferase
Fib-4	Fibrosis-4 score
GGT	Gamma glutamyl transferase
HBsAg	Hepatitis B surface antigen
IAIHG	International Autoimmune Hepatitis Group
IgG	Immunoglobulin G
IQR	Interquartile range
IST	Immunosuppressive therapy
kPa	Kilopascal
MASLD	Metabolic dysfunction-associated steatotic liver disease
MVI	Microvascular imaging
ASMA/SMA	Anti-smooth muscle antibodies
TE	Transient elastography
UDCA	Ursodeoxycholic acid
